# Is Diabetes a Contraindication to Lower Extremity Flap Reconstruction? An Analysis of Threatened Lower Extremities in the NSQIP Database (2010–2020)

**DOI:** 10.1055/a-2233-2617

**Published:** 2024-01-24

**Authors:** Amy Chen, Shannon R. Garvey, Nimish Saxena, Valeria P. Bustos, Emmeline Jia, Monica Morgenstern, Asha D. Nanda, Arriyan S. Dowlatshahi, Ryan P. Cauley

**Affiliations:** 1 Division of Plastic and Reconstructive Surgery, Department of Surgery, Beth Israel Deaconess Medical Center, Harvard Medical School, Boston, Massachusetts

**Keywords:** diabetes, lower extremity, flap reconstruction

## Abstract

**Background** The impact of diabetes on complication rates following free flap (FF), pedicled flap (PF), and amputation (AMP) procedures on the lower extremity (LE) is examined.

**Methods** Patients who underwent LE PF, FF, and AMP procedures were identified from the 2010 to 2020 American College of Surgeons National Surgical Quality Improvement Program (ACS-NSQIP®) database using Current Procedural Terminology and International Classification of Diseases-9/10 codes, excluding cases for non-LE pathologies. The cohort was divided into diabetics and nondiabetics. Univariate and adjusted multivariable logistic regression analyses were performed.

**Results** Among 38,998 patients undergoing LE procedures, 58% were diabetic. Among diabetics, 95% underwent AMP, 5% underwent PF, and <1% underwent FF. Across all procedure types, noninsulin-dependent (NIDDM) and insulin-dependent diabetes mellitus (IDDM) were associated with significantly greater all-cause complication rates compared with absence of diabetes, and IDDM was generally higher risk than NIDDM. Among diabetics, complication rates were not significantly different across procedure types (IDDM: *p* = 0.5969; NIDDM: *p* = 0.1902). On adjusted subgroup analysis by diabetic status, flap procedures were not associated with higher odds of complications compared with amputation for IDDM and NIDDM patients. Length of stay > 30 days was statistically associated with IDDM, particularly those undergoing FF (AMP: 5%, PF: 7%, FF: 14%, *p* = 0.0004).

**Conclusion** Our study highlights the importance of preoperative diabetic optimization prior to LE procedures. For diabetic patients, there were few significant differences in complication rates across procedure type, suggesting that diabetic patients are not at higher risk of complications when attempting limb salvage instead of amputation.

## Introduction

In the aging United States population, diabetes has become increasingly prevalent and costly. 1 2 The impact of diabetes on surgical outcomes is of particular concern as it is associated with a higher risk of complications due to impaired wound healing, disruption of micro- and macrovascular physiology, and altered metabolic response to surgical stress. 3 4 5 6 Diabetes is becoming increasingly common in the population of patients undergoing limb salvage procedures of the lower extremity (LE). 7

In patients with threatened LEs, major types of definitive surgical treatment may include amputation (AMP), pedicled flap (PF) reconstruction, or free tissue transfer. 8 While LE AMP may allow for a good functional outcome in some patients, especially when paired with advances in prosthetics and surgical techniques, it has generally been associated with significant increases in morbidity and mortality, declines in functional status, and exacerbation of comorbid disease. 9 10 11 12 Limb salvage with flap reconstruction is associated with better functional outcomes in most patients, although it may be offered with decreased frequency in patients deemed to have a greater burden of comorbid disease such as diabetes. 9 10 13 14 In an analysis by Gotsman et al of diabetic plastic surgery patients, LE procedures performed on insulin-dependent diabetics were associated with significantly higher odds of wound dehiscence and wound infection than those performed on nondiabetics. 15

Concerns over the potential for complications leading to flap loss and eventual secondary AMP may contribute to hesitancy over deciding whether to proceed with limb salvage or primary AMP. A better understanding of the effect of diabetes on relative complication rates in patients undergoing different major LE reconstructive procedures may guide surgical decision-making, counseling, and resource allocation. The purpose of this study was to examine the adjusted and unadjusted effects of noninsulin- and insulin-dependent diabetes on surgical management, comorbid burden, resource utilization, and complication rates in patients undergoing major reconstructive procedures of the LEs.

## Methods

### Patient Identification

**Fig. 1:**
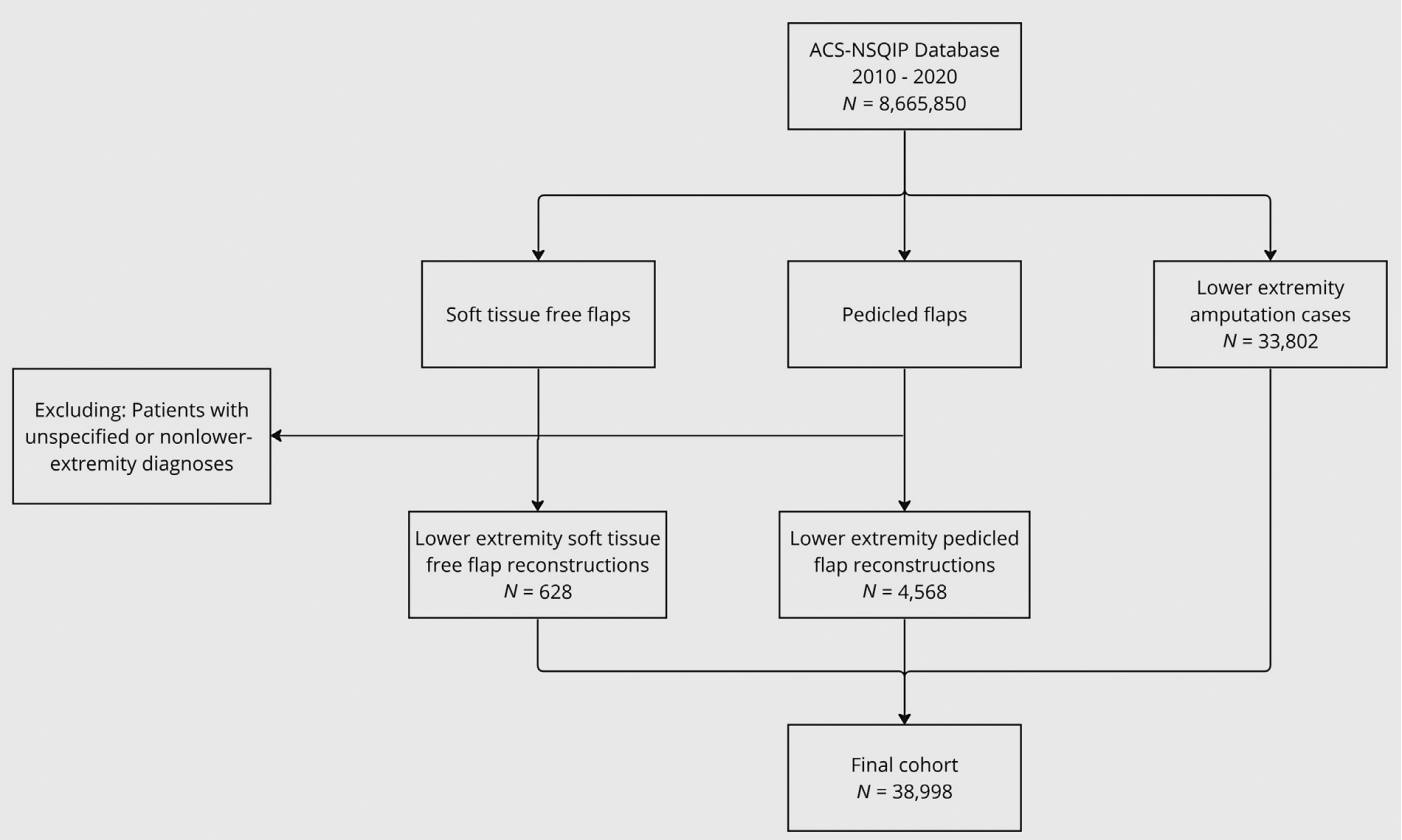
Patient inclusion and exclusion criteria flow chart.

American College of Surgeons National Surgical Quality Improvement Program (ACS-NSQIP) participant data files from 2010 to 2020 were obtained for this study. The database was queried for free flaps (FFs), pedicled muscle, or fasciocutaneous flaps, and AMP above or below the knee using Current Procedural Terminology (CPT) codes ( Table 1 ). More minor procedures such as skin grafting and local tissue rearrangement were excluded if they did not also include one of the above CPT codes. International Classification of Diseases (ICD)-9 and ICD-10 codes were used to identify patients with LE pathologies (from the level of the buttock/sacrum to the toes; Table 2 ). Patients with any primary diagnosis corresponding to an unspecified region or location other than the LE were excluded ( Fig. 1 ).

**Table 1:** Current Procedural Terminology codes and definitions for queried lower extremity procedures

**Free flap**	
15757	Free skin flap with microvascular anastomosis
15758	Free facial flap with microvascular anastomosis
15756	Free flap of muscle and accompanying skin layers with microvascular anastomosis
**Pedicled flap**	
15734	Muscle, myocutaneous, or fasciocutaneous flap elevated from the trunk
15738	Muscle, myocutaneous, or fasciocutaneous flap elevated from the lower extremity
**Amputation**	
27590	AKA with skin and muscle closure
27591	AKA with skin and muscle closure, and casting for immediate prosthesis fitting
27592	Guillotine type AKA with no primary closure
27594	AKA for secondary closure or scar revision
27596	AKA stump revision
27880	BKA with skin flap closure
27881	BKA with skin flap closure, and casting for immediate prosthesis fitting
27882	Guillotine type BKA with no primary closure
27884	BKA for secondary closure or scar revision
27886	BKA stump revision

Abbreviations: AKA, above knee amputation; BKA, below knee amputation; CPT, Current Procedural Terminology.

**Table 2:** International Classification of Diseases-9/10 codes and definitions for selected lower extremity pathologies

***Open wound to the lower extremity***	
**Knee**	
S81.009A	Open wound, unspecified knee, initial encounter
S81.002A	Open wound, left knee, initial encounter
S81.001A	Open wound, right knee, initial encounter
**Lower leg**	
S81.809A	Open wound, unspecified lower leg, initial encounter
S81.802A	Open wound, left lower leg, initial encounter
S81.801A	Open wound, right lower leg, initial encounter
S81.809D	Open wound, unspecified lower leg, subsequent encounter
S81.802D	Open wound, left lower leg, subsequent encounter
S81.801D	Open wound, right lower leg, subsequent encounter
S81.809S	Open wound, unspecified lower leg, sequela
S81.802S	Open wound, left lower leg, sequela
S81.801S	Open wound, right lower leg, sequela
**Ankle**	
S91.009A	Open wound, unspecified ankle, initial encounter
S91.002A	Open wound, left ankle, initial encounter
S91.001A	Open wound, right ankle, initial encounter
S91.009D	Open wound, unspecified ankle, subsequent encounter
S91.002D	Open wound, left ankle, subsequent encounter
S91.001D	Open wound, right ankle, subsequent encounter
S91.009S	Open wound, unspecified ankle, sequela
S91.002S	Open wound, left ankle, sequela
S91.001S	Open wound, right ankle, sequela
**Foot**	
S91.309A	Open wound, unspecified foot, initial encounter
S91.302A	Open wound, left foot, initial encounter
S91.301A	Open wound, right foot, initial encounter
S91.309D	Open wound, unspecified foot, subsequent encounter
S91.302D	Open wound, left foot, subsequent encounter
S91.301D	Open wound, right foot, subsequent encounter
S91.309S	Open wound, unspecified foot, sequela
S91.302S	Open wound, left foot, sequela
S91.301S	Open wound, right foot, sequela
***Acute osteomyelitis***	
**Tibia/fibula**	
M86.169	Other acute osteomyelitis, unspecified tibia and fibula
M86.162	Other acute osteomyelitis, left tibia and fibula
M86.161	Other acute osteomyelitis, right tibia and fibula
M86.069	Acute hematogenous osteomyelitis, unspecified tibia and fibula
M86.062	Acute hematogenous osteomyelitis, left tibia and fibula
M86.061	Acute hematogenous osteomyelitis, right tibia and fibula
**Ankle/foot**	
M86.179	Other acute osteomyelitis, unspecified ankle and foot
M86.172	Other acute osteomyelitis, left ankle and foot
M86.171	Other acute osteomyelitis, right ankle and foot
M86.079	Acute hematogenous osteomyelitis, unspecified ankle and foot
M86.072	Acute hematogenous osteomyelitis, left ankle and foot
M86.071	Acute hematogenous osteomyelitis, right ankle and foot
***Chronic osteomyelitis***	
**Tibia/fibula**	
M86.669	Other chronic osteomyelitis, unspecified tibia and fibula
M86.662	Other chronic osteomyelitis, left tibia and fibula
M86.661	Other chronic osteomyelitis, right tibia and fibula
M86.369	Chronic multifocal osteomyelitis, unspecified tibia and fibula
M86.362	Chronic multifocal osteomyelitis, left tibia and fibula
M86.361	Chronic multifocal osteomyelitis, right tibia and fibula
M86.469	Chronic osteomyelitis with draining sinus, unspecified tibia and fibula
M86.462	Chronic osteomyelitis with draining sinus, left tibia and fibula
M86.461	Chronic osteomyelitis with draining sinus, right tibia and fibula
M86.569	Chronic hematogenous osteomyelitis, unspecified tibia and fibula
M86.562	Chronic hematogenous osteomyelitis, left tibia and fibula
M86.561	Chronic hematogenous osteomyelitis, right tibia and fibula
**Ankle/foot**	
M86.679	Other chronic osteomyelitis, unspecified ankle and foot
M86.672	Other chronic osteomyelitis, left ankle and foot
M86.671	Other chronic osteomyelitis, right ankle and foot
M86.379	Chronic multifocal osteomyelitis, unspecified ankle and foot
M86.372	Chronic multifocal osteomyelitis, left tibia and fibula
M86.371	Chronic multifocal osteomyelitis, right ankle and foot
M86.479	Chronic osteomyelitis with draining sinus, unspecified ankle and foot
M86.472	Chronic osteomyelitis with draining sinus, left ankle and foot
M86.471	Chronic osteomyelitis with draining sinus, right ankle and foot
M86.579	Chronic hematogenous osteomyelitis, unspecified ankle and foot
M86.572	Chronic hematogenous osteomyelitis, left ankle and foot
M86.571	Chronic hematogenous osteomyelitis, right ankle and foot
***Unspecified osteomyelitis***	
**Lower leg**	
M86.8X6	Other osteomyelitis, lower leg

### Demographics and Perioperative Characteristics

Preoperative variables for analysis included demographics (age, gender, race, body mass index [BMI]), and comorbidities (diabetes and insulin use, current smoking status within 1 year of admission, hypertension requiring medication, history of congestive heart failure [CHF], history of chronic obstructive pulmonary disease [COPD], history of renal failure, preoperative steroid use, history of bleeding disorder, 5-Factor Modified Frailty Index [mFI-5] score, American Society of Anesthesiologists [ASA] classification, and ICD-9 and ICD-10 diagnosis codes). The mFI-5 stratifies patients by frailty based on five variables: hypertension requiring medication, diabetes mellitus, history of COPD or pneumonia, functional status, and CHF within 30 days before operation. 16 Primary diagnoses based on ICD-9 and ICD-10 codes were identified and categorized into “Acute, trauma, or orthopaedic” (wound without medical etiology, tendinitis, acute burn, tendon contracture, acute hemorrhage after orthopaedic procedure, idiopathic aseptic necrosis of bone, osteoarthritis, orthopaedic implant failure including acute infection or inflammation), “Chronic or medical etiology” (thromboembolism, chronic infections, chronic osteomyelitis, osteomyelitis unspecified, neurovascular disease from peripheral vascular disease or diabetes, Charcot deformity, complex regional pain syndrome, pressure ulcer), “Malignancy or benign neoplasm,” and “Other or Unknown” (unknown or acquired absence of extremity, failure of skin graft of unknown etiology, unspecified wound).

### Postoperative Outcomes

Postoperative outcomes included wound complications (superficial surgical site infection, deep surgical site infection, organ space surgical site infection, and wound dehiscence), mild systemic complications (pneumonia, bleeding, deep venous thrombosis requiring therapy, sepsis, urinary tract infection, renal insufficiency, and return to operating room), severe systemic complications (pulmonary embolism, unplanned intubation, ventilator use for >48 h, renal failure, cerebrovascular accident, cardiac arrest, myocardial infarction, and septic shock). All-cause complications were defined as having at least one of the above stated complications. Readmission, reoperation, and length of hospital stay greater than 30 days were included as secondary outcome variables.

### Statistical Analysis

Patients were stratified by type of primary procedure (AMP, PF, or FF) and diabetic status (nondiabetic, diabetic on oral medication, diabetic on insulin). Descriptive statistics for preoperative variables and postoperative outcomes were summarized with frequencies with percentages and means with standard deviations for categorical and continuous variables, respectively. For inferential analysis, univariate analysis was performed using the chi-square test for categorical data and one-way analysis of variance test for continuous variables. Multivariate logistic regression models were constructed to determine associations between covariates and all-cause complications. A *p* -value less than or equal to 0.05 was considered significant for all analyses. Statistical analyses were performed using JMP Pro 15.0 (SAS Institute; Cary, NC) and StataBE 17 (StataCorp LLC, College Station, TX).

## Results

A total of 38,998 patients with LE procedures were included in the study. Of those, 86.7% were AMPs, 11.7% were PFs, and 1.6% were FFs. In our cohort of patients undergoing procedures for threatened LEs, AMP rates were significantly higher for patients with diabetes (no diabetes: 75%, noninsulin-dependent diabetes mellitus [NIDDM]: 90%, insulin-dependent diabetes mellitus [IDDM] 96%, *p* < 0.001).

### Stratification of Procedure Type by Diabetic Status: Preoperative Variables

Across all procedure types, univariate analysis demonstrated significant differences between diabetic diagnosis categories in age (AMP: *p* < 0.001; PF, FF: *p* < 0.0001), BMI ( *p* < 0.0001), mFI-5 ( *p* < 0.0001), ASA class ( *p* < 0.0001), and comorbid disease burden. Patients with a diagnosis of NIDDM and/or IDDM trended toward older age, and having higher BMI, higher mFI-5 scores, and higher ASA class. There was also a significant difference in primary diagnosis categories, with diabetic patients more commonly being categorized as having LE wounds with “chronic or medical etiology” ( *p* < 0.0001; Table 3 ).

**Table 3:** Univariate analysis of preoperative variables for amputation, pedicled flap, and free flap patients stratified by diabetic status

	Amputation				Pedicle flap				Free flap			
	Nondiabetic	Diabetic		*p*	Nondiabetic	Diabetic		*p*	Nondiabetic	Diabetic		*p*
		Orals	Insulin			Orals	Insulin			Orals	Insulin	
**Number of patients,** ***n*** **(%)**	12,237 (31.38)	4,806 (12.32)	16,759 (42.97)		3,510 (9.00)	490 (1.26)	568 (1.46)		527 (1.35)	44 (0.11)	57 (0.15)	
**Age, mean ± SD**	66.85 ± 15.23	67.53 ± 12.28	63.70 ± 12.35	<0.001	56.38 ± 16.27	62.2 ± 12.10	61.15 ± 12.16	<0.0001	51.87 ± 17.37	63.50 ± 11.54	56.98 ± 9.35	<0.0001
**Sex,** ***n*** **(%)**				<0.0001				0.9532				0.5271
Male	7,673 (30.32)	3,296 (13.02)	11,240 (44.41)		2,060 (58.69)	293 (59.80)	329 (57.92)		346 (65.65)	32 (72.73)	40 (70.18)	
**BMI, mean ± SD**	25.91 ± 7.13	28.41 ± 7.30	29.89 ± 8.05	<0.0001	28.02 ± 7.01	30.82 ± 7.45	31.22 ± 7.93	<0.0001	27.84 ± 6.24	32.15 ± 9.05	30.71 ± 6.06	<0.0001
**Race,** ***n*** **(%)**				<0.0001				0.0018				0.4184
White	7,711 (63.01)	2,878 (59.88)	10,050 (59.97)		2,667 (75.98)	360 (73.47)	388 (68.31)		377 (71.54)	31 (70.45)	35 (61.40)	
Black or African American	3,150 (25.74)	1,135 (23.62)	4,469 (26.67)		445 (12.68)	65 (13.27)	101 (17.78)		46 (8.73)	5 (11.36)	9 (15.79)	
Other a	1376 (11.24)	793 (16.50)	2,240 (13.37)		398 (11.34)	65 (13.27)	79 (13.91)		104 (19.73)	8 (18.18)	13 (22.81)	
**Current smoker,** ***n*** **(%)**	4,485 (36.65)	1,411 (29.36)	4,009 (23.92)	<0.0001	884 (25.19)	103 (21.02)	135 (23.77)	0.1197	133 (25.24)	7 (15.91)	14 (24.56)	0.385
**5-Factor Modified Frailty Index,** ***n*** **(%)**				<0.0001				<0.0001				<0.0001
0 to 1 points	7,900 (64.56)	530 (11.03)	1,538 (9.18)		3,069 (87.44)	71 (14.49)	71 (12.50)		498 (94.50)	13 (29.55)	8 (14.04)	
2 to 3 points	4,265 (34.85)	4,006 (83.35)	13,903 (82.96)		439 (12.51)	413 (84.29)	476 (83.80)		29 (5.50)	31 (70.45)	49 (85.96)	
4 to 5 points	72 (0.59)	270 (5.62)	1,318 (7.86)		2 (0.06)	6 (1.22)	21 (3.70)		0	0	0	
**ASA Classification,** ***n*** **(%)**				<0.0001				<0.0001				<0.0001
ASA Class 1	45 (0.37)	4 (0.08)	4 (0.02)		76 (2.17)	0	0		35 (6.64)	0	0	
ASA Class 2	832 (6.80)	97 (2.02)	193 (1.15)		922 (26.27)	56 (11.43)	22 (3.87)		243 (46.11)	9 (20.45)	5 (8.77)	
ASA Class 3	6,564 (53.64)	2,871 (59.74)	8,876 (52.96)		2,086 (59.43)	355 (72.45)	379 (66.73)		223 (42.31)	32 (72.73)	43 (75.44)	
ASA Class 4	4,715 (38.53)	1,805 (37.56)	7,585 (45.26)		411 (11.71)	77 (15.71)	163 (28.70)		23 (4.36)	3 (6.82)	8 (14.04)	
ASA Class 5	71 (0.58)	21 (0.44)	78 (0.47)		4 (0.11)	1 (0.20)	2 (0.35)		0	0	1 (1.75)	
**Comorbidities,** ***n*** **(%)**												
Hypertension	8,399 (68.64)	4,044 (84.14)	14,341 (85.57)	<0.0001	1,516 (43.19)	375 (76.53)	443 (77.99)	<0.0001	162 (30.74)	29 (65.91)	42 (73.68)	**<0.0001**
Congestive heart failure	689 (5.63)	334 (6.95)	1,490 (8.89)	<0.0001	39 (1.11)	9 (1.84)	30 (5.28)	<0.0001	3 (0.57)	0	1 (1.75)	0.4856
COPD	1,640 (13.40)	438 (9.11)	1,752 (10.45)	<0.0001	230 (6.55)	37 (7.55)	59 (10.39)	0.0041	20 (3.80)	3 (6.82)	5 (8.77)	0.1645
Renal failure	318 (2.60)	140 (2.91)	939 (5.60)	<0.0001	11 (0.31)	3 (0.61)	11 (1.94)	<0.0001	0	0	2 (3.51)	<0.0001
Preoperative steroid use	830 (6.78)	172 (3.58)	986 (5.88)	<0.0001	135 (3.85)	23 (4.69)	48 (8.45)	<0.0001	20 (3.80)	0	5 (8.77)	0.0708
Bleeding disorder	2,317 (7.58)	901 (2.95)	3,278 (10.72)	0.319	216 (0.71)	49 (0.16)	85 (0.28)	<0.0001	18 (0.06)	1 (0.00)	8 (0.03)	0.0005
**ICD-9 and ICD-10 diagnosis codes**												
** Categories,** ***n*** **(%)**				<0.0001				<0.0001				<0.0001
Acute, trauma, or orthopaedic b	563 (4.60)	97 (2.02)	221 (1.32)		716 (20.40)	108 (22.04)	120 (21.13)		155 (29.41)	6 (13.64)	6 (10.53)	
Chronic or medical etiology c	10,060 (82.21)	4,241 (88.24)	14,561 (86.88)		1747 (49.77)	258 (52.65)	355 (62.50)		128 (24.29)	20 (45.45)	32 (56.14)	
Malignancy/benign neoplasm d	129 (1.05)	27 (0.56)	17 (0.10)		695 (19.80)	72 (14.69)	27 (4.75)		100 (18.98)	5 (11.36)	5 (8.77)	
Other or unknown e	1,485 (12.14)	441 (9.18)	1,960 (11.70)		352 (10.03)	52 (10.61)	66 (11.62)		144 (27.32)	13 (29.55)	14 (24.56)	

Abbreviations: %, column percentage; ASA, American Society of Anesthesiologists; BMI, body mass index; COPD, chronic obstructive pulmonary disease; ICD, International Classification of Diseases; *n* , frequency; SD, standard deviation.

a Includes Asian, Native Hawaiian or Pacific Islander, and Native Hawaiian or Other Pacific Islander.

b Includes ICD-9 and ICD-10 codes corresponding to the following: wound without medical etiology, tendinitis, acute burn, tendon contracture, acute hemorrhage after orthopaedic procedure, idiopathic aseptic necrosis of bone, osteoarthritis, orthopaedic implant failure including acute infection or inflammation.

c Includes ICD-9 and ICD-10 codes corresponding to the following: thromboembolism, chronic infections, chronic osteomyelitis, osteomyelitis unspecified, neurovascular disease from peripheral vascular disease or diabetes, Charcot deformity, complex regional pain syndrome, pressure ulcer.

d Includes ICD-9 and ICD-10 codes corresponding to the following: malignancy, benign neoplasm.

e Includes ICD-9 and ICD-10 codes corresponding to the following: unknown or acquired absence of extremity, failure of skin graft of unknown etiology, unspecified wound.

### Stratification of Diabetic Status by Procedure Type: Preoperative Variables

Of the total patient sample, 41.7% were nondiabetics, 13.7% were NIDDM, and 44.6% were IDDM. Across diabetes diagnosis categories, patients receiving different procedure types differed significantly by age ( *p* < 0.0001), BMI ( *p* < 0.0001), race ( *p* < 0.0001), burden of comorbid disease, by frailty index ( *p* < 0.0001), and ASA class ( *p* < 0.0001). Within NIDDM and IDDM groups, patients who received PF or FF procedures trended toward younger age, higher BMI, White race, lower frailty index, and lower ASA class. Among nondiabetics and NIDDM, patients undergoing flap reconstruction were significantly less likely to be current smokers ( *p* < 0.0001). Across diabetes diagnosis categories, flap patients tended to have primary diagnoses categorized as “Acute, Trauma, or Orthopaedic,” “Malignancy/Benign Neoplasm,” or “Other or Unknown” etiology ( Table 4 ).

**Table 4:** Univariate analysis of preoperative variables for nondiabetic, noninsulin-dependent diabetic, and insulin-dependent diabetic patients stratified by procedure type

	*Nondiabetic*				*Diabetic on oral medication*				*Diabetic on insulin*			
	*Amp.*	*Pedicle*	*Free flap*	*p*	*Amp.*	*Pedicle*	*Free flap*	*p*	*Amp.*	*Pedicle*	*Free flap*	*p*
**Number of patients,** ***n (%)***	12,237 (75.19)	3,510 (21.57)	527 (3.24)		4,806 (90.00)	490 (9.18)	44 (0.82)		16,759 (96.40)	568 (3.27)	57 (0.33)	
**Age,** ***mean ± SD***	66.85 ± 12.23	56.38 ± 16.27	51.87 ± 17.37	<0.0001	67.53 ± 12.28	62.2 ± 12.10	63.50 ± 11.54	<0.0001	63.70 ± 12.35	61.15 ± 12.16	56.98 ± 9.35	<0.0001
**Gender,** ***n (%)***				<0.0001				0.0019				0.0003
Male	7,673 (62.70)	2,060 (58.69)	346 (65.65)		3,296 (68.58)	293 (59.80)	32 (72.73)		11,240 (67.07)	329 (57.92)	40 (70.18)	
**BMI,** ***mean ± SD***	25.91 ± 7.13	28.02 ± 7.01	27.84 ± 6.24	<0.0001	28.41 ± 7.30	30.82 ± 7.45	32.15 ± 9.05	<0.0001	29.89 ± 8.05	31.22 ± 7.93	30.71 ± 6.06	0.0005
**Race,** ***n (%)***				<0.0001				<0.0001				<0.0001
White	7,711 (63.01)	2,667 (75.98)	377 (71.54)		2,878 (59.88)	360 (73.47)	31 (70.45)		10,050 (59.97)	388 (68.31)	35 (61.40)	
Black or African American	3,150 (25.74)	445 (12.68)	46 (8.73)		1,135 (23.62)	65 (13.27)	5 (11.36)		4,469 (26.67)	101 (17.78)	9 (15.79)	
Other a	1,376 (11.24)	398 (11.34)	104 (19.73)		793 (16.50)	65 (13.27)	8 (18.18)		2,240 (13.37)	79 (13.91)	13 (22.81)	
**Current smoker,** ***n (%)***	4,485 (36.65)	884 (25.19)	133 (25.24)	<0.0001	1,411 (29.36)	103 (21.02)	7 (15.91)	<0.0001	4,009 (23.92)	135 (23.77)	14 (24.56)	0.99
**5-Factor Modified Frailty Index**				<0.0001				<0.0001				<0.0001
0 to 1 Points	7,900 (64.56)	3,069 (87.44)	498 (94.50)		530 (11.03)	71 (14.49)	13 (29.55)		1,538 (9.18)	71 (12.50)	8 (14.04)	
2 to 3 Points	4,265 (34.85)	439 (12.51)	29 (5.50)		4,006 (83.35)	413 (84.29)	31 (70.45)		13,903 (82.96)	476 (83.80)	49 (85.96)	
4 to 5 Points	72 (0.59)	2 (0.06)	0		270 (5.62)	6 (1.22)	0		1,318 (7.86)	21 (3.70)	0	
**ASA classification,** ***n (%)***				<0.0001				<0.0001				<0.0001
ASA Class 1	45 (0.37)	76 (2.17)	35 (6.64)		4 (0.08)	0	0		4 (0.02)	0	0	
ASA Class 2	832 (6.80)	922 (26.27)	243 (46.11)		97 (2.02)	56 (11.43)	9 (20.45)		193 (1.15)	22 (3.87)	5 (8.77)	
ASA Class 3	6,564 (53.64)	2,086 (59.43)	223 (42.31)		2,871 (59.74)	355 (72.45)	32 (72.73)		8,876 (52.96)	379 (66.73)	43 (75.44)	
ASA Class 4	4,715 (38.53)	411 (11.71)	23 (4.36)		1,805 (37.56)	77 (15.71)	3 (6.82)		7,585 (45.26)	163 (28.70)	8 (14.04)	
ASA Class 5	71 (0.58)	4 (0.11)	0		21 (0.44)	1 (0.20)	0		78 (0.47)	2 (0.35)	1 (1.75)	
**Comorbidities** ***n (%)***												
Hypertension	8,399 (68.64)	1,516 (43.19)	162 (30.74)	<0.0001	4,044 (84.14)	375 (76.53)	29 (65.91)	<0.0001	14,341 (85.57)	443 (77.99)	42 (73.68)	<0.0001
Congestive heart failure	689 (5.63)	39 (1.11)	3 (0.57)	<0.0001	334 (6.95)	9 (1.84)	0	<0.0001	1,490 (8.89)	30 (5.28)	1 (1.75)	0.002
COPD	1,640 (13.40)	230 (6.55)	20 (3.80)	**<0.0001**	438 (9.11)	37 (7.55)	3 (6.82)	0.4539	1,752 (10.45)	59 (10.39)	5 (8.77)	0.9167
Renal failure	318 (2.60)	11 (0.31)	0	**<0.0001**	140 (2.91)	3 (0.61)	0	**0.0059**	939 (5.60)	11 (1.94)	2 (3.51)	0.0006
Preoperative steroid use	830 (6.78)	135 (3.85)	20 (3.80)	**<0.0001**	172 (3.58)	23 (4.69)	0	0.1966	986 (5.88)	48 (8.45)	5 (8.77)	0.0268
Bleeding disorder	2,317 (7.58)	216 (0.71)	18 (0.06)	**<0.0001**	901 (2.95)	49 (0.16)	1 (0.00)	**<0.0001**	3,278 (10.72)	85 (0.28)	8 (0.03)	0.0499
**ICD-9 and ICD-10 diagnosis codes**												
**Categories,** ***n (%)***				**<0.0001**				**<0.0001**				<0.0001
Acute, trauma, or orthopaedic b	563 (4.60)	716 (20.40)	155 (29.41)		97 (2.02)	108 (22.04)	6 (13.64)		221 (1.32)	120 (21.13)	6 (10.53)	
Chronic or medical etiology c	10,060 (82.21)	1,747 (49.77)	128 (24.29)		4,241 (88.24)	258 (52.65)	20 (45.45)		14,561 (86.88)	355 (62.50)	32 (56.14)	
Malignancy/benign neoplasm d	129 (1.05)	695 (19.80)	100 (18.98)		27 (0.56)	72 (14.69)	5 (11.36)		17 (0.10)	27 (4.75)	5 (8.77)	
Other or unknown e	1,485 (12.14)	352 (10.03)	144 (27.32)		441 (9.18)	52 (10.61)	13 (29.55)		1,960 (11.70)	66 (11.62)	14 (24.56)	

Abbreviations: %, column percentage; Amp., amputation; ASA, American Society of Anesthesiologists; BMI, body mass index; COPD, chronic obstructive pulmonary disease; ICD, ; *n* , frequency; SD, standard deviation.

a Includes Asian, Native Hawaiian or Pacific Islander, and Native Hawaiian or Other Pacific Islander.

b Includes ICD-9 and ICD-10 codes corresponding to the following: wound without medical etiology, tendinitis, acute burn, tendon contracture, acute hemorrhage after orthopaedic procedure, idiopathic aseptic necrosis of bone, osteoarthritis, orthopaedic implant failure including acute infection or inflammation.

c Includes ICD-9 and ICD-10 codes corresponding to the following: thromboembolism, chronic infections, chronic osteomyelitis, osteomyelitis unspecified, neurovascular disease from peripheral vascular disease or diabetes, Charcot deformity, complex regional pain syndrome, pressure ulcer.

d Includes ICD-9 and ICD-10 codes corresponding to the following: malignancy, benign neoplasm.

e Includes ICD-9 and ICD-10 codes corresponding to the following: unknown or acquired absence of extremity, failure of skin graft of unknown etiology, unspecified wound.

### Stratification of Procedure Type by Diabetic Status: Outcomes

Increasing rates of all-cause complications were significantly associated with diabetic status and insulin-dependency across all treatment types: AMP ( *p* < 0.0001), PF ( *p* < 0.0001), and FF patients ( *p* = 0.0500). Patients with a diagnosis of diabetes tended to have a greater rate of mild systemic complications, whether they were undergoing an AMP ( *p* < 0.0001), a PF ( *p* < 0.0001), or a FF ( *p* = 0.0629). Severe systemic complications were highest among IDDM patients who underwent AMPs ( *p* = 0.0159) and PFs ( *p* < 0.0001), and were not significantly associated with diabetic status for FF patients ( *p* = 0.5725). Wound complications were not significantly associated with diabetic status for any procedure type.

Readmission and reoperation rates were not significantly associated with diabetic status in FF patients. Rates of length of stay (LOS) greater than 30 days were significantly different across diabetic diagnosis categories among FF patients ( *p* = 0.0024), and highest for IDDM patients. LOS greater than 30 days and was not associated with diabetic status for AMP and PF patients ( Table 5 ).

**Table 5:** Univariate analysis of postoperative outcomes for amputation, pedicled flap, and free flap patients stratified by diabetic status

	*Amputation*				*Pedicle flap*				*Free flap*			
	*Nondiabetic*	*Diabetic*		*p*	*Nondiabetic*	*Diabetic*		*p*	*Nondiabetic*	Diabetic		*p*
		*Orals*	*Insulin*			*Orals*	*Insulin*			Orals	Insulin	
**Number of patients,** ***n (%)***	12,237 (31.38)	4,806 (12.32)	16,759 (42.97)	–	3,510 (9.00)	490 (1.26)	568 (1.46)	–	527 (1.35)	44 (0.11)	57 (0.15)	–
** All-cause complications, ab** ***n (%)***	4,541 (37.11)	1,871 (38.93)	7,194 (42.93)	<0.0001	1095 (31.20)	180 (36.73)	253 (44.54)	<0.0001	171 (32.45)	12 (27.27)	27 (47.37)	**0.0500**
** Wound complications, ac** ***n (%)***	1,074 (8.87)	404 (8.41)	1,377 (8.22)	0.2367	497 (14.16)	72 (14.69)	92 (16.20)	0.4356	71 (13.47)	4 (9.09)	13 (22.81)	0.0968
** Mild systemic complications, ad** ***n (%)***	3,348 (27.26)	1,421 (29.57)	5,623 (33.55)	<0.0001	732 (20.85)	121 (24.69)	184 (32.39)	<0.0001	128 (24.29)	11 (25.00)	22 (38.60)	0.0629
Pneumonia	528 (4.31)	153 (3.18)	659 (3.93)	0.0029	68 (1.94)	6 (1.22)	16 (2.82)	0.1704	7 (1.33)	1 (2.27)	1 (1.75)	0.8597
Deep venous thrombosis	128 (1.05)	33 (0.66)	139 (0.83)	0.1080	40 (1.14)	1 (0.20)	7 (1.24)	0.4077	7 (1.33)	1 (2.27)	1 (1.75)	0.8597
Bleeding	2,306 (18.84)	1,022 (21.27)	4,070 (24.29)	<0.0001	500 (14.25)	83 (16.94)	136 (23.94)	<0.0001	97 (18.41)	10 (22.73)	16 (28.07)	0.1876
Sepsis	595 (4.86)	254 (5.29)	1,075 (6.41)	<0.0001	181 (5.16)	35 (7.14)	45 (7.92)	0.011	20 (3.80)	1 (2.27)	7 (12.28)	**0.0099**
Urinary tract infection	346 (2.83)	141 (2.93)	521 (3.11)	0.3717	70 (1.99)	12 (2.45)	14 (2.46)	0.6544	5 (0.95)	0	1 (1.75)	0.6672
Renal insufficiency	69 (0.56)	44 (0.92)	174 (1.04)	<0.0001	19 (0.54)	3 (0.61)	6 (1.06)	0.345	1 (0.19)	0	2 (3.51)	**0.0023**
** Severe systemic complications, ae** ***n (%)***	1,257 (10.27)	473 (9.84)	1,856 (11.07)	0.0159	135 (3.85)	18 (3.67)	51 (8.98)	<0.0001	15 (2.85)	1 (2.27)	3 (5.26)	0.5725
Pulmonary embolism	60 (0.49)	27 (0.56)	54 (0.32)	0.022	19 (0.54)	1 (0.20)	0	0.1371	3 (0.57)	0	0	0.7491
Unplanned intubation	340 (2.78)	113 (2.35)	506 (3.02)	0.0432	38 (1.08)	5 (1.02)	19 (3.35)	<0.0001	5 (0.95)	1 (2.27)	1 (1.75)	0.6445
Ventilation use for >48 hours	313 (2.56)	92 (1.91)	408 (2.43)	0.0448	54 (1.54)	4 (0.82)	21 (3.70)	0.0003	2 (0.38)	1 (2.27)	1 (1.75)	0.1706
Renal failure	84 (0.69)	48 (1.00)	216 (1.29)	**<0.0001**	12 (0.34)	1 (0.20)	3 (0.53)	0.6629	0	0	2 (3.51)	**<0.0001**
Stroke/CVA	83 (0.68)	36 (0.75)	122 (0.73)	0.8395	8 (0.23)	1 (0.20)	2 (0.35)	0.8417	0	0	0	–
Cardiac arrest	202 (1.65)	87 (1.81)	392 (2.34)	**0.0001**	18 (0.51)	3 (0.61)	8 (1.41)	**0.0046**	0	0	1 (1.75)	0.0066
Myocardial infarction	163 (1.33)	101 (2.10)	336 (2.00)	**<0.0001**	12 (0.34)	6 (1.22)	10 (1.76)	**<0.0001**	2 (0.38)	0	0	0.8251
Septic shock	338 (2.76)	104 (2.16)	450 (2.69)	0.0788	46 (1.31)	3 (0.61)	15 (2.64)	**0.0127**	4 (0.76)	0	0	0.6799
**Length of stay > 30 days,** ***n (%)***	539 (4.40)	209 (4.35)	796 (4.75)	0.2797	180 (5.13)	22 (4.49)	39 (6.87)	0.1625	21 (3.98)	1 (2.27)	8 (14.04)	0.0024
**Readmission,** ***n (%)***	1570 (13.89)	553 (12.44)	2369 (15.13)	**<0.0001**	343 (10.13)	44 (9.28)	84 (15.61)	**0.0004**	44 (8.64)	1 (2.33)	6 (10.71)	0.2873
**Reoperation,** ***n (%)***	1013 (8.96)	343 (7.72)	1249 (7.98)	**0.0050**	303 (8.95)	33 (6.96)	72 (13.38)	**0.0008**	79 (15.52)	4 (9.30)	14 (25.00)	0.0860

Abbreviations: %, column percentage; CVA, cerebrovascular accident; *n* , frequency.

a Aggregates of complications reflect the number of patients with at least one complication and thus this figure is not equal to the sum of the individual components.

b At least one of the following: all variables included in wound, mild systemic, or severe systemic complications.

c At least one of the following: superficial surgical site infection, deep surgical site infection, organ space surgical site infection, or wound dehiscence.

d At least one of the following: pneumonia, bleeding, deep venous thrombosis requiring therapy, sepsis, urinary tract infection, renal insufficiency, return to operating room.

e At least one of the following: pulmonary embolism, unplanned intubation, ventilator use for >48 hours, renal failure, stroke/CVA, cardiac arrest, myocardial infarction, septic shock.

### Stratification of Diabetic Status by Procedure Type: Outcomes

All-cause complications were significantly higher for AMPs than pedicled or FF reconstructions among nondiabetics ( *p* < 0.0001). For patients with IDDM or NIDDM, all-cause complication rates were significantly higher than those for nondiabetics ( Table 5 ). Nevertheless, there were no significant differences in all-cause complication rates across procedure types among patients with IDDM ( *p* = 0.5969) or NIDDM ( *p* = 0.1902; Table 6 ). Wound complication rates were higher following flap reconstruction cases than AMPs for nondiabetics ( *p* < 0.0001), NIDDM ( *p* < 0.0001), and IDDM ( *p* < 0.0001). Severe systemic complications were highest following AMPs for both nondiabetics ( *p* < 0.0001) and NIDDM ( *p* = 0.0192). For patients with IDDM, mild systemic complications, severe systemic complications, and readmission rates were not significantly different across procedure types. Rates of reoperation ( *p* < 0.0001) and LOS greater than 30 days ( *p* = 0.0004) were both highest following FF procedures on IDDM patients ( Table 6 ).

**Table 6:** Univariate analysis of postoperative outcomes for nondiabetic, noninsulin-dependent diabetic, and insulin-dependent diabetic patients stratified by procedure type

	*Nondiabetic*				*Diabetic on oral medication*				*Diabetic on insulin*			
	*Amp.*	*Pedicle*	*Free flap*	*p*	*Amp.*	*Pedicle*	*Free flap*	*p*	*Amp.*	*Pedicle*	*Free flap*	*p*
**Number of patients,** ***n*** **(%)**	12,237 (75.19)	3,510 (21.57)	527 (3.24)	–	4,806 (90.00)	490 (9.18)	44 (0.82)	–	16,759 (96.40)	568 (3.27)	57 (0.33)	–
** All-cause complications, ab** ***n*** **(%)**	4,541 (37.11)	1095 (31.20)	171 (32.45)	<0.0001	1,871 (38.930)	180 (36.73)	12 (27.27)	0.1902	7,194 (42.93)	253 (44.54)	27 (47.37)	0.5969
** Wound complications, ac** ***n*** **(%)**	1,074 (8.87)	497 (14.16)	71 (13.47)	<0.0001	404 (8.41)	72 (14.69)	4 (9.09)	<0.0001	1,377 (8.22)	92 (16.20)	13 (22.81)	<0.0001
** Mild systemic complications, ad** ***n*** **(%)**	3,348 (27.26)	732 (20.85)	128 (24.29)	<0.0001	1,421 (29.57)	121 (24.69)	11 (25.00)	0.0646	5,623 (33.55)	184 (32.39)	22 (38.60)	0.6099
Pneumonia	528 (4.31)	68 (1.94)	7 (1.33)	<0.0001	153 (3.18)	6 (1.22)	1 (2.27)	0.0510	659 (3.93)	16 (2.82)	1 (1.75)	0.2829
Deep venous thrombosis	128 (1.05)	40 (1.14)	7 (1.33)	0.8241	33 (0.66)	1 (0.20)	1 (2.27)	0.3968	139 (0.83)	7 (1.24)	1 (1.75)	0.7438
Bleeding	2,306 (18.84)	500 (14.25)	97 (18.41)	<0.0001	1,022 (21.27)	83 (16.94)	10 (22.73)	0.0769	4,070 (24.29)	136 (23.94)	16 (28.07)	0.7866
Sepsis	595 (4.86)	181 (5.16)	20 (3.80)	**0.3839**	254 (5.29)	35 (7.14)	1 (2.27)	0.1459	1,075 (6.41)	45 (7.92)	7 (12.28)	0.0731
Urinary tract infection	346 (2.83)	70 (1.99)	5 (0.95)	**0.0013**	141 (2.93)	12 (2.45)	0	0.4308	521 (3.11)	14 (2.46)	1 (1.75)	0.5768
Renal insufficiency	69 (0.56)	19 (0.54)	1 (0.19)	0.5213	44 (0.92)	3 (0.61)	0	0.6496	174 (1.04)	6 (1.06)	2 (3.51)	0.1876
Severe systemic complications, a e *n* (%)	1,257 (10.27)	135 (3.85)	15 (2.85)	**<0.0001**	473 (9.84)	18 (3.67)	1 (2.27)	**<0.0001**	1,856 (11.07)	51 (8.98)	3 (5.26)	0.1117
Pulmonary embolism	60 (0.49)	19 (0.54)	3 (0.57)	0.9103	27 (0.56)	1 (0.20)	0	0.5156	54 (0.32)	0	0	0.3642
Unplanned intubation	340 (2.78)	38 (1.08)	5 (0.95)	**<0.0001**	113 (2.35)	5 (1.02)	1 (2.27)	0.1641	506 (3.02)	19 (3.35)	1 (1.75)	0.7735
Ventilation use for >48 hours	313 (2.56)	54 (1.54)	2 (0.38)	**<0.0001**	92 (1.91)	4 (0.82)	1 (2.27)	0.2168	408 (2.43)	21 (3.70)	1 (1.75)	0.1531
Renal failure	84 (0.69)	12 (0.34)	0	**0.0126**	48 (1.00)	1 (0.20)	0	0.1738	216 (1.29)	3 (0.53)	2 (3.51)	0.0901
Stroke/CVA	83 (0.68)	8 (0.23)	0	**0.0015**	36 (0.75)	1 (0.20)	0	0.3281	122 (0.73)	2 (0.35)	0	0.4708
Cardiac arrest	202 (1.65)	18 (0.51)	0	**<0.0001**	87 (1.81)	3 (0.61)	0	0.0997	392 (2.34)	8 (1.41)	1 (1.75)	0.3348
Myocardial infarction	163 (1.33)	12 (0.34)	2 (0.38)	**<0.0001**	101 (2.10)	6 (1.22)	0	0.2659	336 (2.00)	10 (1.76)	0	0.5144
Septic shock	338 (2.76)	46 (1.31)	4 (0.76)	**<0.0001**	104 (2.16)	3 (0.61)	0	0.0416	450 (2.69)	15 (2.64)	0	0.4548
**Length of stay > 30 days,** ***n (%)***	539 (4.40)	180 (5.13)	21 (3.98)	**0.1583**	209 (4.35)	22 (4.49)	1 (2.27)	0.7868	796 (4.75)	39 (6.87)	8 (14.04)	**0.0004**
**Readmission** ***n (%)***	1570 (13.89)	343 (10.13)	44 (8.64)	**<0.0001**	553 (12.44)	44 (9.28)	1 (2.33)	**0.0192**	2,369 (15.13)	84 (15.61)	6 (10.71)	0.6225
**Reoperation** ***n (%)***	1013 (8.96)	303 (8.95)	79 (15.52)	**<0.0001**	343 (7.72)	33 (6.96)	4 (9.30)	0.7742	1,249 (7.98)	72 (13.38)	14 (25.00)	**<0.0001**

Abbreviations: %, column percentage; Amp., amputation; CVA, cerebrovascular accident; *n* , frequency.

a Aggregates of complications reflect the number of patients with at least one complication and thus this figure is not equal to the sum of the individual components.

b At least one of the following: all variables included in wound, mild systemic, or severe systemic complications.

c At least one of the following: superficial surgical site infection, deep surgical site infection, organ space surgical site infection, or wound dehiscence.

d At least one of the following: pneumonia, bleeding, deep venous thrombosis requiring therapy, sepsis, urinary tract infection, renal insufficiency, return to operating room.

e At least one of the following: pulmonary embolism, unplanned intubation, ventilator use for >48 hours, renal failure, stroke/CVA, cardiac arrest, myocardial infarction, septic shock.

### Multivariable Analysis

Compared with nondiabetic patients who underwent either AMP or limb salvage procedures, patients with IDDM had a 1.207 greater odds of having any complication after adjusting for potential confounders (95% confidence interval [CI] 1.149–1.269, *p* < 0.001). Other independent risk factors for an increased odds of all-cause complications included patients undergoing a FF, a chronic wound diagnosis, older age, female sex, Black race, higher BMI, dependent functional status, presence of CHF, and diagnosis of hypertension ( Table 7 ). No significant interaction was found between NIDDM and PFs ( *p* = 0.8738), or between NIDDM and FFs ( *p* = 0.1863). Furthermore, there were no significant interaction effects found between IDDM and PFs ( *p* = 0.2116), or between IDDM and FFs ( *p* = 0.1604)—suggesting that patients with a diagnosis of diabetes (NIDDM or IDDM) are not subject to a relatively increased risk of complications when undergoing flaps compared with nondiabetics. Subgroup multivariate analysis demonstrated that while FFs were associated with a significantly increased adjusted odds of all-cause complications in nondiabetics, in subsets of both NIDDM and IDDM there were no significantly elevated adjusted odds of all-cause complications associated with pedicled and FFs ( Tables 8 9 10 ).

**Table 7:** Multivariable regression analysis of all-cause complications based on diabetic status, procedure, International Classification of Diseases codes, and risk factors

	*Adjusted odds of all-cause complications*		*p* -Value
	*OR*	*95% CI*	
*Diabetes*			
No diabetes	*Reference*		
Noninsulin-dependent diabetes	1.037	0.969–1.110	0.2922
Insulin-dependent diabetes a	1.207	1.149–1.269	**<0.0001**
**Procedure**			
Amputation	*Reference*		
Pedicle flap	0.999	0.925–1.079	0.9865
Free flap	1.197	1.003–1.429	**0.0458**
**ICD-9 and ICD-10 diagnosis codes**			
Malignancy/benign neoplasm b	*Reference*		
Acute, trauma, or orthopaedic c	1.449	1.224–1.716	**<0.0001**
Chronic or medical etiology d	1.610	1.383–1.874	**<0.0001**
Other or unknown e	1.075	0.914–1.264	0.3832
**Age**			
	*1.009*	*1.006* – *1.012*	**0.0006**
**Gender**			
Female	*Reference*		
Male	0.932	0.892–0.974	**0.0019**
**Race**			
White	*Reference*		
Black or African American	*1.123*	*1.070* – *1.181*	**<0.0001**
Other f	*1.085*	*1.015* – *1.159*	**0.0165**
**BMI**			
	*1.009*	*1.006* – *1.012*	**<0.0001**
**Functional status**			
Nondependent	*Reference*		
Dependent	*1.397*	*1.335* – *1.462*	**<0.0001**
**CHF**			
	*1.682*	*1.549* – *1.826*	**<0.0001**
**HTN**			
	*1.133*	*1.073* – *1.196*	**<0.0001**

Abbreviations: 95% CI, 95% confidence interval; BMI, body mass index; CHF, congestive heart failure; HTN, hypertension; ICD, International Classification of Diseases; OR, odds ratio.

a No significant interactions between insulin-dependency and pedicle flaps ( *p* = 0.2116), and between insulin-dependency and free flaps ( *p* = 0.1604).

b Includes ICD-9 and ICD-10 codes corresponding to the following: thromboembolism, chronic infections, chronic osteomyelitis, osteomyelitis unspecified, neurovascular disease from peripheral vascular disease or diabetes, Charcot deformity, complex regional pain syndrome, pressure ulcer.

c Includes ICD-9 and ICD-10 codes corresponding to the following: wound without medical etiology, tendinitis, acute burn, tendon contracture, acute hemorrhage after orthopaedic procedure, idiopathic aseptic necrosis of bone, osteoarthritis, orthopaedic implant failure including acute infection or inflammation.

d Includes ICD-9 and ICD-10 codes corresponding to the following: malignancy, benign neoplasm.

e Includes ICD-9 and ICD-10 codes corresponding to the following: unknown or acquired absence of extremity, failure of skin graft of unknown etiology, unspecified wound.

f Includes Asian, Native Hawaiian or Pacific Islander, and Native Hawaiian or Other Pacific Islander.

**Table 8:** Multivariable regression analysis for all-cause complications among nondiabetic patients

	Adjusted odds of all-cause complications a		*p* -Value
	OR	95% CI	
**No diabetes**			
Amputation	Reference		
Pedicled flap	1.020	0.928–1.120	0.6874
Free flap	1.343	1.100–1.639	**0.0037**

Abbreviations: 95% CI, 95% confidence interval; OR, odds ratio.

a Adjusted for International Classification of Diseases (ICD)-9 and ICD-10 diagnosis codes, age, gender, race, body mass index, functional status, congestive heart failure, and hypertension requiring medication.

**Table 9:** Multivariable regression analysis for all-cause complications among noninsulin-dependent diabetic patients

	Adjusted odds of all-cause complications a		*p* -Value
	OR	95% CI	
**Noninsulin-dependent diabetes**			
Amputation	Reference		
Pedicled flap	1.049	0.841–1.309	0.6708
Free flap	0.776	0.394–1.527	0.4631

Abbreviations: 95% CI, 95% confidence interval; OR, odds ratio.

a Adjusted for International Classification of Diseases (ICD)-9 and ICD-10 diagnosis codes, age, gender, race, body mass index, functional status, congestive heart failure, and hypertension requiring medication.

**Table 10:** Multivariable regression analysis for all-cause complications among insulin-dependent diabetic patients

	Adjusted odds of all-cause complications a		*p* -Value
	OR	95% CI	
**Insulin-dependent diabetes**			
Amputation	Reference		
Pedicle flap	1.059	0.884–1.268	0.5340
Free flap	1.281	0.747–2.194	0.3679

Abbreviations: 95% CI, 95% confidence interval; OR, odds ratio.

a Adjusted for International Classification of Diseases (ICD)-9 and ICD-10 diagnosis codes, age, gender, race, body mass index, functional status, congestive heart failure, and hypertension requiring medication.

## Discussion

LE wounds in diabetic patients remains challenging for providers and has placed significant burdens on the health care system at-large. There is ongoing debate over whether diabetic patients presenting with LE wounds should undergo limb salvage rather than AMP, due to concerns over diabetic comorbidities that may compromise wound healing following flap reconstruction. This study seeks to provide a more complete understanding of the precise effects of diabetes on surgical management, comorbid burden, resource utilization, and complication rates in patients undergoing major reconstructive procedures of the LEs.

To our knowledge, this study is the first to utilize a national database to compare the independent effects of IDDM and NIDDM on patients undergoing AMPs, PFs, and FF interventions for the treatment of LE pathologies. Our analysis confirms several previously held findings in the literature and in clinical practice: (1) that although diabetic patients received operative intervention for threatened LEs more frequently than nondiabetics, diabetic patients were less likely to receive LE flap reconstruction than AMP, and (2) diabetics tend to have higher complication rates than nondiabetics, and IDDM tended to have higher complication rates than NIDDM. 17 18 19 20 Importantly, we found that among patients with NIDDM and IDDM, there was no difference in adjusted complication rates across procedure types (AMP, PFs, and free tissue transfer), suggesting that diabetic patients are not at higher risk of complications when attempting limb salvage instead of AMP.

In all patients, the decision to attempt limb salvage with the use of a soft tissue reconstruction such as a pedicled or FF requires careful consideration of individual factors including significant comorbidities. 21 Free tissue transfers, which are important in cases where there are no locoregional options, may be further complicated by recipient vessels that are calcified, stenosed, or adjacent to a chronically infected wound. Concomitant peripheral vascular disease affecting flow distal to the recipient vessel may also raise concerns of postoperative distal tissue perfusion and ischemia. Diabetic patients have been demonstrated to have both a greater burden of comorbid disease and to be at higher risk of complications when undergoing almost any type of surgical procedure. In microvascular reconstruction, elevated blood glucose levels increase the risk of surgical site infections, wound dehiscence, and fistulas. Given this increased risk, many surgeons may be hesitant to offer limb salvage in patients with diabetes, especially if it is more severe or poorly controlled. Indeed, despite the considerable utility of FFs in diabetic LE wounds, they are undertaken infrequently and limited to highly specialized centers—therefore highlighting an unmet need. 14

The rate of diabetes within our sample of patients was 58.3% (44.6% IDDM, 13.7% NIDDM), which is significantly higher than that of the general population. This is in agreement with data from prior studies, which have demonstrated that diabetic patients are overrepresented in the surgical population. 4 We found that in this large, national patient sample, both pedicled and FF reconstructions were significantly less common in patients with a diagnosis of diabetes. Furthermore, patients with IDDM were less likely to undergo a flap reconstruction compared with patients with NIDDM—presumably given the greater severity of disease in the insulin-dependent patient population. Among both diabetic and nondiabetics, patients who received AMPs were generally older, were most likely to have LE pathology due to chronic medical disease, and had lower BMI, higher frailty, higher ASA classification, and higher rates of comorbidities compared with patients who received PFs and FF reconstruction ( Table 4 ). Among AMP patients, advanced diabetes—indicated by insulin-dependency—was associated with the highest frailty scores and comorbidity rates ( Table 3 ). The opposite trend was observed for patients who received FFs and did not have diabetes. This cohort was found to be the healthiest; they were most likely to have LE wounds from trauma or orthopaedic pathology, and had the lowest frailty scores and comorbidity rates ( Table 3 ). Furthermore, no FF patients, regardless of their diabetic status, had frailty scores higher than 3 points ( Table 3 ). These observations likely reflect a simple selection bias, as surgeons are presumably more likely to offer complex microsurgical reconstruction to healthier patients.

As expected from previous literature, all-cause complication rates and mild systemic complication rates were found to be higher for diabetic patients than nondiabetic patients across all procedure types ( Table 5 ). 18 20 Insulin-dependency further increased each of these risks compared with diabetics on oral medications alone. However, a major finding of our analysis is that in both the IDDM and NIDDM cohorts, complication rates for patients undergoing PFs or free tissue transfer were not significantly higher than those undergoing AMP ( Table 6 ). Only nondiabetics were consistently found to have significant differences in complication rates according to procedure type ( Table 6 ). These observations in our univariate analysis indicate that patients with more advanced diabetes had similarly high rates of complications regardless of whether they underwent AMP, PF, or FF procedures. Multivariate analysis further supports this finding. Overall, IDDM was found to independently predict higher all-cause complications (odds ratio [OR] 1.207, 95% CI 1.149–1.269, *p* < 0.001). In the overall cohort, free tissue transfer was also independently associated with a higher odds of all-cause complications (OR 1.197, 95% CI 1.003–1.429, *p* = 0.046) compared with AMP. However, when only examining patients with IDDM and NIDDM diagnosis on subgroup analysis, we found that flap procedures were not associated with significantly higher odds of complications compared with AMPs ( Tables 9 and 10 ). Furthermore, multivariate analysis demonstrated no significant interaction term between diabetes and flap procedures ( Table 7 )—suggesting that although diabetes may generally increase complication rates, neither NIDDM nor IDDM increase the relative risks of attempting a PF or a free tissue transfer instead of an AMP.

A similar trend was also observed for severe systemic complication and readmission rates for IDDM patients: within the AMP and PF groups, IDDM patients had the highest rates of severe systemic complications and readmissions, but among IDDM patients there were no significant differences across procedure types ( Tables 5 and 6 ). This again suggests that patients with advanced diabetes had elevated complication rates regardless of whether they underwent AMP or limb salvage, and that these complications rates did not significantly differ based on procedure type.

Wound complication rates were higher for flap procedures than AMP, regardless of diabetic status—likely due to donor site morbidity, larger incisions, and longer operating times associated with flap reconstruction ( Table 6 ). This may have contributed to higher reoperation rates observed for FFs among both NIDDM and IDDM patients ( Table 6 ). LOS >30 days was significantly higher for IDDM patients who received FFs, likely due to prolonged postoperative stay to monitor flap viability ( Table 6 ).

Overall, we found that for patients with more advanced or poorly controlled diabetes, outcomes are expected to be equivocal irrespective of whether the patient undergoes limb salvage or AMP. We conclude, therefore, that diabetes alone should not discourage surgeons from choosing free or PF reconstruction over AMP for threatened LEs. Ideally, diabetic patients should undergo optimization of glycemic control prior to surgery, and the degree to which this is achieved should inform surgical planning. For more urgent cases however, such medical optimization may not be feasible. In these circumstances, our data suggest that patients with poorer glycemic control and/or more advanced disease may have similar risk for complications regardless of whether they undergo AMP, PF, or FF procedures. We postulate that for diabetic patients with a threatened limb, the decision to amputate or salvage the limb with a pedicled or FF may have less of an effect on the subsequent complication rate than other factors such as age, functional status, and other comorbidities. We suspect that for surgical planning in diabetic patients, greater consideration should be placed on factors such as the location and size of the wound, neurovascular status, potential need for reoperation, direct and indirect financial costs to the patient, time required for postoperative recovery, and the wishes of the patient, than the presumed complication rate. Further prospective studies should be conducted comparing these factors among diabetic patients receiving AMP, PF, and FF interventions for LE wounds.

### Limitations

While the results of this study demonstrated differences and trends in postoperative outcomes according to insulin-dependency of diabetic patients, this analysis did not include HbA1c (hemoglobin A1c) because the variable is not tracked by the NSQIP database. Although transition from oral hypoglycemics to insulin broadly represents rising insulin resistance and an advancement in overall disease progression, HbA1c provides a more accurate indication of glycemic control in the preoperative period—a patient with IDDM may have tighter glycemic control due to good regimen adherence, whereas a patient with NIDDM may have suboptimal glycemic control due to nonadherence. However, we believe that this potential limitation would not substantially detract from the findings and conclusions of our study, as prior literature has demonstrated that both HbA1c and glycated albumin levels show greater variability among IDDM patients than NIDDM patients. 22 Nevertheless, future studies of the effect of A1c on complication rates in patients undergoing LE reconstruction will be needed.

As the NSQIP database only differentiates diabetic patients by insulin use, IDDM includes both type 1 and type 2 diabetic patients, which likely contributed to the much higher rates of IDDM in the sample (44.6% IDDM, 13.7% NIDDM). While the inclusion of type 1 diabetics in the IDDM sample could also potentially complicate our use of insulin-dependency as an indicator of advanced disease, type 1 diabetics are most likely a minority in this sample. In a recent population study of adults with diabetes in the United States, the weighted percentages of type 1 and type 2 diabetes were reported to be 5 to 6% and 90 to 92%, respectively. 23 As expected, the study also demonstrated type 2 diabetes was more prevalent among older and obese adults, which correlates with findings for the IDDM sample in our analysis. Therefore, our data demonstrate that the majority of the IDDM sample is likely composed of type 2 diabetic patients who have progressed to insulin-dependency.

This study is also subject to other limitations of the NSQIP database. Patients were identified for this study through CPT and ICD codes, and errors in diagnosis and coding may therefore affect the accuracy of the presented data. Patients with missing data could also affect accuracy, but variables included in the study did not have significantly large portions of missing data, and were therefore deemed suitable for analysis. Furthermore, although this study found that diabetic patients underwent AMP more frequently than nondiabetic patients, it is important to note that other factors such as severity of other comorbidities, surgeon skill, and hospital resource availability, may all affect the patient selection process and cannot be accounted for by the database.

We also note that since the NSQIP database only tracks postoperative complications up to the 30th day after surgery, this study only provides a cross-sectional analysis that does not account for complications that may occur outside the immediate postoperative period. In addition, complications related to flap survival are not specified in the database, nor can complications be linked to certain procedures performed for each patient case. Outcomes reported in the analyses are therefore based on incidence within a small postoperative window and not necessarily on prevalence, preventing this study from providing any conclusions on long-term outcomes. Finally, other than measuring 30-day complication rates collected in the NSQIP database, this study is unable to determine the success or failure of each of these procedures.

### Conclusion

This study examines the impact of diabetes mellitus on postoperative outcomes in patients who underwent AMP, PF, or FF procedures for LE pathologies in one of the largest published cohorts of LE wound patients. Analysis of this large national cohort confirmed previous findings that diabetes is independently associated with an increased risk of all-cause complications. This increase in complication risk was found to be stepwise, with insulin-dependency being associated with higher risk than noninsulin-dependency. Both these points suggest the importance of glycemic control and medical optimization prior to limb salvage. While FF procedures were independently associated with an increased odds of complications compared with AMPs in the overall patient cohort, patients with insulin- and noninsulin-dependent diabetes had similar postoperative complication rates regardless of whether they underwent limb salvage with flap procedures or AMP. Given that diabetic patients are at equally high risk across procedure types, a diagnosis of diabetes should not necessarily discourage a surgeon from choosing limb salvage over flap reconstruction.
